# Improved maximum likelihood method for P-S-N curve fitting method with small number specimens and application in T-welded joint

**DOI:** 10.1038/s41598-023-46594-y

**Published:** 2023-11-06

**Authors:** Wenfei Liu, Li Zhang, Liwen He, Hailang Liu

**Affiliations:** 1 School of Intelligent Manufacture, Taizhou University, Taizhou, 318000 China; 2 Baotou Beifang Chuangye Co., Ltd, Baotou, 014032 China

**Keywords:** Mechanical properties, Metals and alloys, Mechanical engineering, Computational methods

## Abstract

In fatigue data analysis, fitting accurate P-S-N curve is problematic if only a small number of specimen is available, especially to evaluate the relationship between the stress level and the standard deviation. This paper proposes a sample information reconstruction method that can effectively solve this problem. Based on this method and the life equivalent principle, a new maximum likelihood method (which is abbreviated to improved maximum likelihood method) is proposed for P-S-N curve fitting. T-joint specimens of Q450NQR1 steel were fabricated and tested, then the P-S-N curves was fitted by the improved maximum likelihood method, least square method, maximum likelihood method, standard BS7608 and standard IIW. Finally, P-S-N curves by three methods and two standards are compared and analyzed. The results show that the relevant parameters of the P-S-N curve with 99.9% survival probability fitted by the improved maximum likelihood method are similar to those in the two standards, and it is indicated that the improved maximum likelihood method is a better way for P-S-N curve fitting with the small number of fatigue test specimens.

## Introduction

P-S-N(probabilistic stress-life) curve is used to describe the relationship between stress level and the number of stress cycles to material fatigue failure for a specified survival probability^1–3^. It is a key indicator of the material’s ability to resist fatigue damage and also the basis for structural fatigue reliability design and evaluation^4–7^. The basic methods used for P-S-N curve fitting include: the least-square method, Bayesian method, maximum likelihood method, Monte Carlo method, and modern intelligent algorithm^8–10^. In fact, each P-S-N curve is fitted with one of methods listed above or a combination of these methods^9, 11–13^.

According to sampling theory, the larger the sample, the higher the accuracy of the tests conducted on the sample. However, owing to high costs and long fatigue testing times, it is difficult to achieve sample sizes that satisfy sampling theory requirements, especially in the case of large-sized structural parts or expensive parts^14–16^. The maximum likelihood method can fit the P-S-N curve with a small sample^17, 18^, but the life means and standard deviations under each stress level must have been measured with high accuracy to achieve good results. Often, fatigue test data exhibit the following pattern^19, 20^: the samples have a small number of specimens in the middle life zone, there is generally a good linear relationship between the mean life and stress level in the logarithmic coordinate system, and the relationship between the life standard deviation and stress level is often not unique. Thus, it is often difficult to reflect the true-life distribution when the number of specimens is small.

In this paper, the sample information reconstruction and the improved maximum likelihood method are studied under small number specimens. Then fatigue test is carried out on T-joint specimens of Q450NQR1 steel, and the P-S-N curves were fitted by the method in this paper, least square method, maximum likelihood method, standard BS7608 and standard IIW. Finally, the P-S-N curves by three methods and two standards are compared and analyzed.

## Theoretical background

### Mathematical expression of S–N curve

The S–N curve represents the fatigue performance of a material. For ease of calculation, mathematical expressions are usually used to describe its laws, including the power function, exponential function, three-parameter, Basquin, and Weibull formulas^21–23^. The S–N curve for a metallic material can be expressed by the power function formula in middle life:

1 $$S^{m} N = C_{0}$$

where* S* is the stress level, *N* is the material life under stress level *S*, *m* and *C* can be determined by the means of a regression of formula (1), and are related to the material, stress ratio, loading mode, and so forth.

Taking the logarithm of both sides in Eq. (1) leads to:

2 $${\text{lg}}S = - \frac{1}{m}{\text{lg}}N + \frac{{{\text{lg}}C_{0} }}{m}$$

Letting $${\text{Y}} = {\text{lg}}S$$, $${\text{X}} = {\text{lg}}N$$, $${\text{A}} = - {\raise0.7ex\hbox{$1$} \!\mathord{\left/ {\vphantom {1 m}}\right.\kern-0pt} \!\lower0.7ex\hbox{$m$}}$$, and $$B = {\raise0.7ex\hbox{${{\text{lg}}C_{0} }$} \!\mathord{\left/ {\vphantom {{{\text{lg}}C_{0} } m}}\right.\kern-0pt} \!\lower0.7ex\hbox{$m$}}$$, Eq. (2) can be rewritten as:

3 $$Y = AX + B$$

Equation (3) shows that it is a linear relationship between the fatigue life *N* and stress level *S* in the logarithmic coordinate system.

### Basic assumption

A large number of fatigue tests have confirmed that the fatigue life for a metallic material generally follows a lognormal distribution in across the mid-life span^24, 25^. Let the number of stress levels be $n$, the total number of specimens be $k$*,* the number of specimens at the *i-th* stress level be ${k}_{i}$, and the fatigue life of the *j-th* specimen at the *i-th* stress level be ${X}_{ij}$. Then the probability density function can be expressed as:

4 $$f\left( {X_{ij} } \right) = \frac{1}{{\sigma_{i} \sqrt {2\pi } }}{\text{exp}}\left[ { - \frac{1}{2}\left( {\frac{{X_{ij} - \mu_{i} }}{{\sigma_{i} }}} \right)^{2} } \right],$$

where $i=\mathrm{1,2},\dots ,n$, $j=\mathrm{1,2},\dots ,{k}_{i}$, and ${\mu }_{i}$ and ${\sigma }_{i}$ are the mean and std (standard deviation) of the log-life (logarithmic fatigue life) under stress level ${S}_{i},$ respectively.

The probability distribution function is:

5 $$F\left( {X_{ij} } \right) = P(X < X_{ij} ) = \Phi \left( {\frac{{X_{ij} - \mu_{i} }}{{\sigma_{i} }}} \right)$$

### Life equivalent principle

From Eq. (1), when the loading method, loading form, stress ratio and test method are determined, the fatigue life of specimen is determined by material performance and manufacturing quality. During fatigue test, it is impossible to perform fatigue test on the same specimen at different stress levels, but according to “consistency principle of fatigue life percentile”^26^, the life equivalent of a specimen at different stress levels can be achieved because the fatigue life probability distribution $F({X}_{ij})$ of the same specimen is constant under different stress levels in medium life span. Thus, the *j-th* specimen’s fatigue life at the *i-th* stress level can be obtained by using Eq. (6) below:

6 $$\frac{{X_{ij} - \mu_{i} }}{{\sigma_{i} }} = T\left( {T\,{\text{is a constant,}}\,i = {1},{2}, \, \ldots ,n} \right)$$

Based on this method, the number of specimens can be increased for different stress levels, maximizing sample information. In particular, it provides a good way to expand the number of specimens. However, this method is based on obtaining a highly accuracy mean life and life std for each stress level. In fact, the mean life and life std are related to the stress level. Therefore, it is especially important to find the relationship between mean life and stress level as well as the relationship between life std and stress level.

### P-S-N curve fitting based on the maximum likelihood method

The basic idea of the maximum likelihood method is that the probability of an event that has occurred is a maximum. Therefore, the selection of unknown parameters is based on the benefit of that event. When using the maximum likelihood method to fit the P-S-N curve, fatigue tests are performed on one specimen at each stress level ${S}_{i}$ (*i* = 1, 2, …, *n*), resulting in a corresponding log-life of ${X}_{i}$. Then a group of specimens are tested under one specified stress level ${S}_{d}$, where the corresponding log-lives are ${X}_{dj}$ (*j* = 1, 2, …, *q*)^27^. Meanwhile, two assumptions are proposed for the mid-life span: the fatigue lives follow a lognormal distribution at each stress level, and the life mean and life std each have a linear relationship with stress level in the logarithmic coordinate system. Then, the following formula can be obtained

7 $$\mu_{i} = \mu_{d} + \alpha \left( {Y_{i} - Y_{d} } \right)$$

8 $$\sigma_{i} = \sigma_{d} + \beta \left( {Y_{i} - Y_{d} } \right),$$

where ${Y}_{i}$ and ${Y}_{d}$ are the values of ${S}_{i}$ and ${S}_{d}$, respectively, in the logarithmic coordinate system; ${\mu }_{d}$ and ${\sigma }_{d}$ are the log-life mean and log-life std, respectively of the sample at stress level ${S}_{d}$; and $\alpha$ and $\beta$ are pending constants.

The log-life with failure probability *p* is:

9 $$X_{p} = \mu + u_{p} \sigma$$

According to the above assumptions, the likelihood function can be written as:

10 $$L = \mathop \prod \limits_{i = 1}^{n} f\left( {X_{i} } \right) = \mathop \prod \limits_{i = 1}^{n} \frac{1}{{\sigma_{i} \sqrt {2\pi } }}\exp \left[ { - \frac{1}{2}\left( {\frac{{X_{i} - \mu_{i} }}{{\sigma_{i} }}} \right)^{2} } \right]$$

Substituting Eqs. (7) and (8) into Eq. (10) and taking the logarithm of both sides of Eq. (10) leads to:

11 $$lnL = - \mathop \sum \limits_{i = 1}^{n} \left\{ {ln\sqrt {2\pi } + ln\left[ {\sigma_{d} + \beta \left( {Y_{i} - Y_{d} } \right)} \right] + \frac{1}{2}\left( {\frac{{X_{i} - \mu_{d} - \alpha \left( {Y_{i} - Y_{d} } \right)}}{{\sigma_{d} + \beta \left( {Y_{i} - Y_{d} } \right)}}} \right)^{2} } \right\}$$

When $lnL$ reaches its maximum value, the corresponding $\alpha$ and $\beta$ reach their maximum likelihoods. If we take partial derivatives of Eq. (11) and set them equal to zero, we can find their values numerically instead.

12 $$\frac{\partial lnL}{{\partial \alpha }} = - \mathop \sum \limits_{i = 1}^{n} \left\{ {\frac{{\left( {Y_{i} - Y_{d} } \right)[X_{i} - \mu_{d} - \alpha \left( {Y_{i} - Y_{d} } \right)]}}{{\left[ {\sigma_{d} + \beta \left( {Y_{i} - Y_{d} } \right)} \right]^{2} }}} \right\} = 0$$

13 $$\frac{\partial lnL}{{\partial \beta }} = - \mathop \sum \limits_{i = 1}^{n} \left\{ {\frac{{\left( {Y_{i} - Y_{d} } \right)[X_{i} - \mu_{d} - \alpha \left( {Y_{i} - Y_{d} } \right)]^{2} }}{{\left[ {\sigma_{d} + \beta \left( {Y_{i} - Y_{d} } \right)} \right]^{3} }} + \frac{{\left( {Y_{i} - Y_{d} } \right)}}{{\left[ {\sigma_{d} + \beta \left( {Y_{i} - Y_{d} } \right)} \right]}}} \right\} = 0$$

Using the above method, the P-S-N curve’s equation is:

14 $$X_{p} = \mu_{d} + u_{p} \sigma_{d} + \left( {\alpha + u_{p} \beta } \right)\left( {Y_{i} - Y_{d} } \right)$$

Equation (14) can be rewritten as:

15 $$Y_{i} = \frac{{X_{p} - \mu_{d} - u_{p} \sigma_{d} }}{{\left( {\alpha + u_{p} \beta } \right)}} + Y_{d}$$

## Improved maximum likelihood method for P-S-N curve fitting

### Sample information reconstruction method

The problem with fitting the P-S-N curve using the maximum likelihood method is that the mean life and life std for each stress level cannot be obtained, with the exception of stress level ${S}_{d}$. Meanwhile, due to high costs and the long time periods needed for fatigue tests, it is difficult to use large samples for fatigue tests. Therefore, this research focuses on obtaining the mean life and life std for each stress level with high accuracy based on small or very small samples.

**Figure 1:**
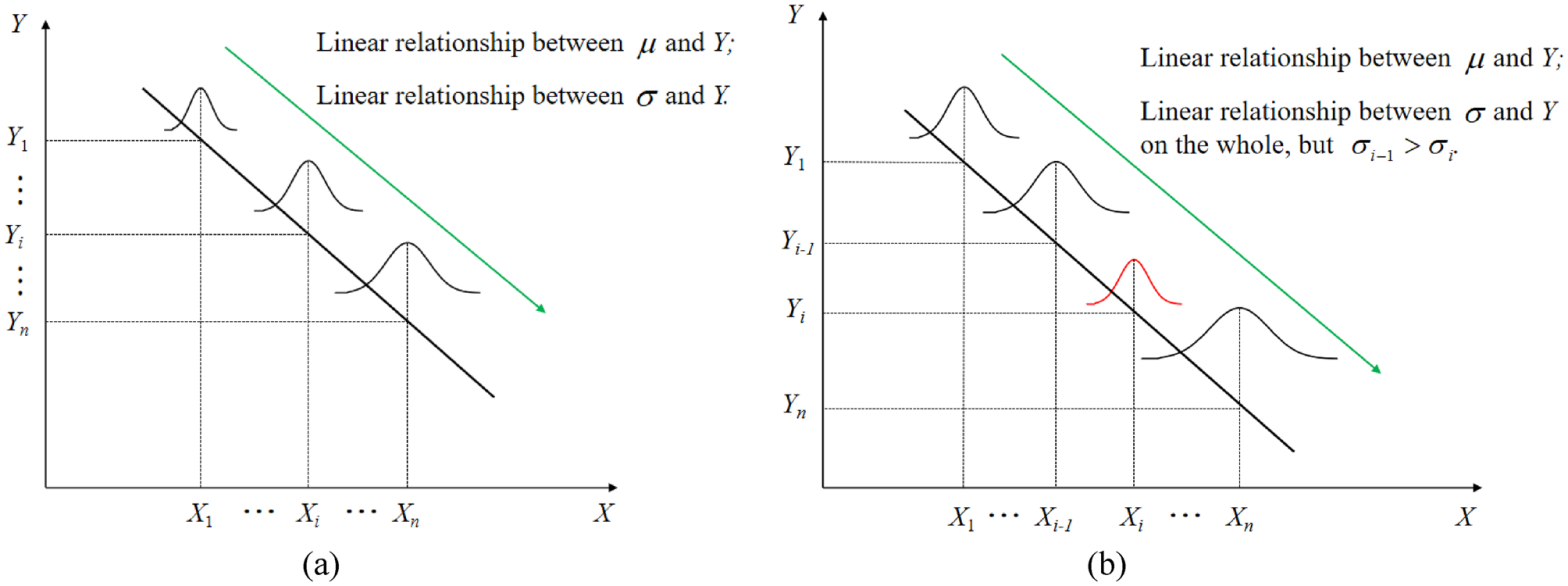
Fatigue life distributions when there is (**a**) a large number of specimens and (**b**) a small number of specimens.

Generally speaking, when the number of specimens for each stress level is large enough, there is an approximate linear relationship between the $\mu$ (or $\sigma$) and stress level in the logarithmic coordinate, which is shown in Fig. 1a. However, in the case of small or very small number samples, it is difficult for fatigue test data to accurately describe true data distribution pattern, particularly in the case of the relationship between stress level and the life std.

In fact, the most typical phenomenon is that they are not linear relationship in some adjacent stress levels when the number of samples is small, which is shown in Fig. 1b. There may even be a phenomenon that it is the opposite of the homoskedasticity method^28^ or linear heteroskedasticity method^29^. The sample information reconstruction method is given to deal with such problems in this paper.

When the fatigue life stds’ change direction (increase or decrease) of two adjacent stress levels is inconsistent with the overall trend, it can combine the two stress levels into one stress level, and Eq. (16) can be defined under the new stress level, as follows:

16 $$\left\{ \begin{gathered} S_{i}^{\prime} = \frac{{k_{i} }}{k}S_{i} + \frac{{k_{i + 1} }}{k}S_{i + 1} \hfill \\ k = k_{i} + k_{i + 1} \hfill \\ \sigma_{i}^{\prime} = \frac{{k_{i} }}{k}\sigma_{i} + \frac{{k_{i + 1} }}{k}\sigma_{i + 1} \hfill \\ \end{gathered} \right.,$$

where ${S}_{i}^{\prime}$ is the new stress level replacing the old stress levels ${S}_{i}$ and ${S}_{i+1}$, ${\sigma }_{i}{\prime}$ is the new life std replacing the old life stds ${\sigma }_{i}$ and ${\sigma }_{i+1}$, and $k$ is the sum of ${k}_{i}$ and ${k}_{i+1}$.

When the fatigue life stds’ change directions (increase or decrease) of three adjacent stress levels relationships are inconsistent with the overall trend, these three stress levels can be combined into one stress level, and Eq. (17) below can be defined under the new stress level, where its parameters are defined as in Eq. (16).

17 $$\left\{ \begin{gathered} S_{i}^{\prime} = \frac{{k_{i - 1} }}{k}S_{i - 1} + \frac{{k_{i} }}{k}S_{i} + \frac{{k_{i + 1} }}{k}S_{i + 1} \hfill \\ k = k_{i - 1} + k_{i} + k_{i + 1} \hfill \\ \sigma_{i}^{\prime} = \frac{{k_{i - 1} }}{k}\sigma_{i - 1} + \frac{{k_{i} }}{k}\sigma_{i} + \frac{{k_{i + 1} }}{k}\sigma_{i + 1} \hfill \\ \end{gathered} \right.$$

Sample information equivalence shall be performed as this method if the variation is not unique between life mean and stress level, and this method is defined as sample information reconstruction.

### Improved maximum likelihood method and its calculation process

**Figure 2:**
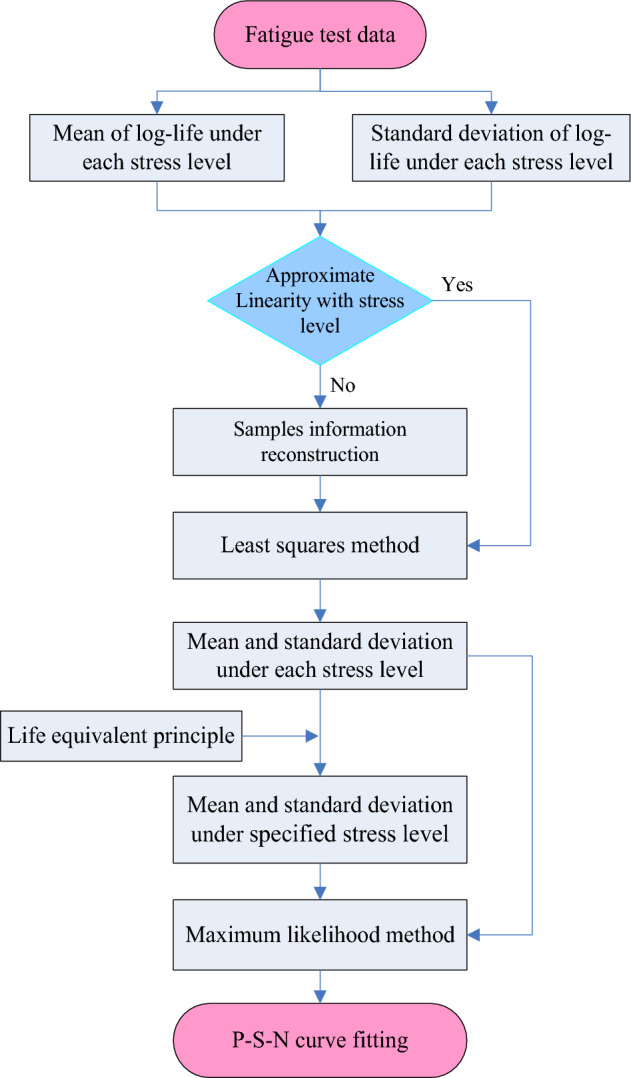
The calculation flowchart of improved maximum likelihood method.

The basic principle of the improved maximum likelihood method is: Firstly, the test data are compiled using the life equivalent principle method, which, when combined with the sample information reconstruction method, can both improve the accuracy of the life stds and ensure the accuracy of the life equivalency. Secondly, the P-S-N curve is fitted via the maximum likelihood method. The calculation flowchart of improved maximum likelihood method for P-S-N curve fitting is shown in Fig. 2, and the detailed steps for the method are as follows:

1. According to the fatigue test data, if there is an approximate linear relationship between the mean life and stress level in logarithmic coordinates at each stress level, then those lines can be fitted by the least squares method, and a mean life mean can be calculated for each stress level.
2. If there is an approximate linear relationship on the whole between life std and stress level in the logarithmic coordinate system but some adjacent stress levels fail to follow the trend closely, the sample information reconstruction method can be used to generate new data, allowing the life stds at different stress levels to be calculated.
3. According to the life equivalent principle, combined with the new means and the new stds, the fatigue lives under different stress levels are equivalent to those at the specific stress level ${S}_{d}$ for a large sample, so all the information in the existing sample is utilized.
4. Finally, the P-S-N curve is fitted with this equivalent fatigue data via the maximum likelihood method.

## Fatigue test of T-joint specimen

Welded T-joints in railway wagons are widely used to provide the basic parameters for fatigue life evaluations of these wagons and can be used to verify the accuracy of the P-S-N curve fitted via the new maximum likelihood method. Thus, fatigue tests were carried out on 30 T-joint specimens made of Q450NQR1 steel, then the P-S-N curve was fitted.

### Material properties

At present, the total number of railway wagons in China exceeds 900,000, and their structures are basically all-steel welded^30^. Q450NQR1 steel is the most widely used. Therefore, it is very representative to fabricate fatigue specimens out of Q450NQR1 steel. Tables 1 and 2 show the chemical composition and mechanical properties of Q450NQR1, respectively.

**Table 1:** Chemical composition of Q450NQR1 steel ^31^.

Element (%)	C	Si	Mn	S	Cr	Ni	Cu
Q450NQR1	≤ 0.12	≤ 0.75	≤ 1.50	≤ 0.008	0.30–1.25	0.12–0.65	0.20–0.55

**Table 2:** Mechanical properties of Q450NQR1 steel ^31^.

Performance index	Lower yield strength (MPa)	Tensile strength (MPa)	Elongation after fracture (%)	Impact property (J)
Q450NQR1	450	550	20	60

### Specimen fabrication

**Figure 3:**
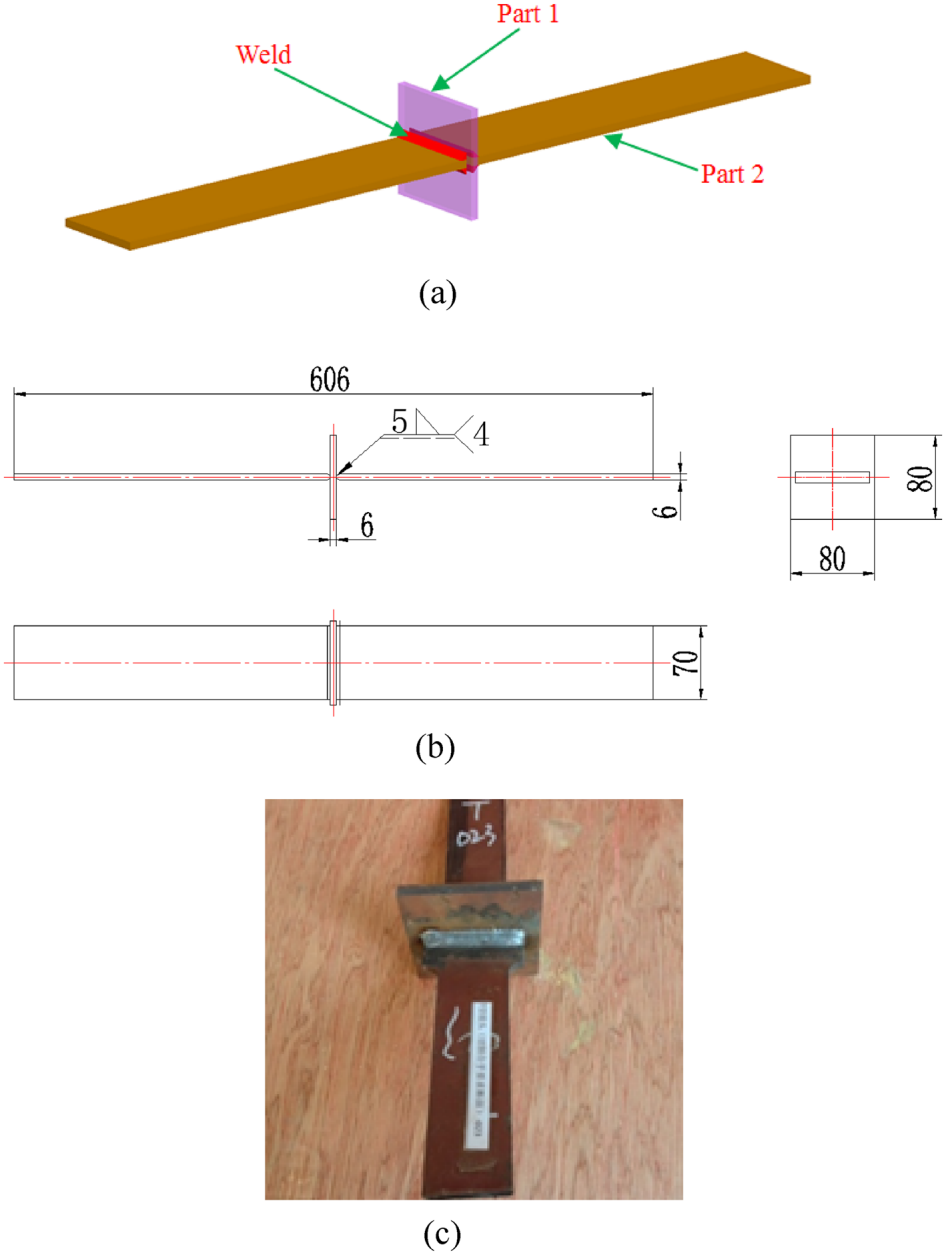
Welded T-joint specimen: (**a**) three dimensional model; (**b**) 2D drawing; (**c**) actual specimen.

Specimen sizes for the fatigue tests followed Standard GB/3075-2008^32^. For each specimen, the thickness of the plate was 6 mm, the dimensional tolerance was ± 1 mm, the vertical tolerance was ± 1 mm, the flatness was within 1 mm, and the parallelism of each side was less than 0.2 mm. The specimens had no defects, such as delamination, depressions, and large bulges, but fine burrs were allowed. The welding method of parts adopts mixed gas shielded welding, the mixed gas ratio is Ar:CO_2_ = 80%:20%, the gas flow is 15–20 L/min; the welding voltage is 18–23 V, and the welding current is 150–200A. A total of 30 T-joint specimens were fabricated. The welded form, specimen size and an actual specimen are shown in Fig. 3.

### Loading conditions

At present, the S–N curves of metallic materials are usually obtained by group test method and lifting method^33^, and the fatigue test in this paper also adopts this methods. The fatigue tests were carried out with the Zwick/Roell high-frequency fatigue testing machine under normal temperatures. The specimens were loaded with a multi-level, constant-amplitude loading consisting of no less than five stages. The loading method utilized was the axial pull-pull cycle with a stress ratio R = 0.1, sine wave load, and loading frequency of about 70 Hz. In order to obtain a realistic fatigue limit, the load stress was adjusted as close as possible to the stress level at a fatigue life of 2 × 10^6^, which was then used as the basis for judging the fatigue failure of the specimen. That is, if the number of cycles exceeded 2 × 10^6^, the specimen was no longer subject to damage.

### Test results

**Figure 4:**
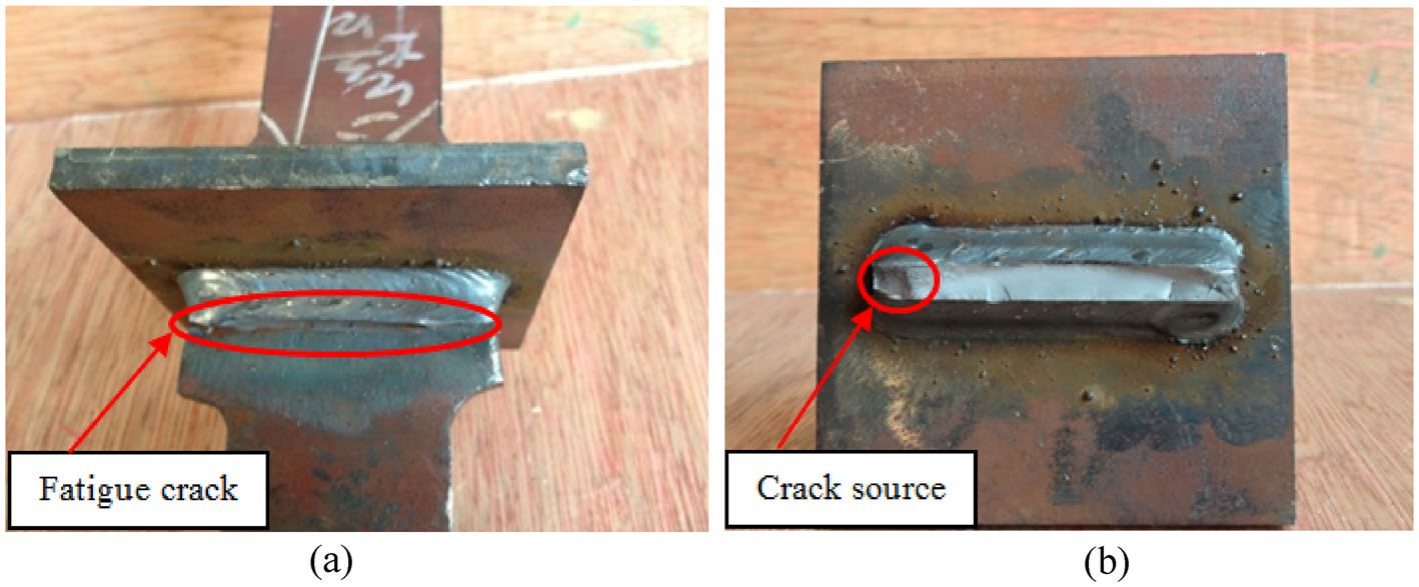
The fatigue test results for a T-joint specimen: (**a**) its fatigue crack and; (**b**) its macroscopic fracture morphology.

The fatigue test results show that the fatigue cracks of all specimens are basically located at the weld toe, and the cracks spread laterally along the position of the weld toe, which is shown in Fig. 4. According to Fig. 4b, the fatigue crack began on the T-joint surface. A fatigue fracture is described by its source, fatigue crack stability extension, and rapid fracture zones. In the figure, the fatigue source zone is located at the end of the T-joint, and the crack growth rate is slower in this area. The upper and lower surfaces continuously opened and closed during the expansion process, leading to little friction, as it is relatively smooth. The white area in the middle of the fracture is the rapid fracture zone, which is a fresh section that appears after a crack has spread sufficiently far.

The fatigue test results for the specimens were calculated. For P-S-N curve fitting, the specimen fatigue life was logarithmically transformed, and the means and stds at the different stress levels were calculated. The results are shown in Table 3.

**Table 3:** The log-lives of T-joint specimens.

Maximum nominal stress/MPa	Log-life	Mean	Std
150	5.4706, 5.4709, 5.4772, 5.515, 5.5437	5.4955	0.0327
140	5.6502, 5.6626, 5.6946, 5.6998	5.6768	0.0242
130	5.7467, 5.8074, 5.8607	5.8049	0.057
120	5.8724, 5.9375, 5.9609	5.9236	0.0458
110	5.9909, 5.9997, 6.0472, 6.1222, 6.1482	6.0616	0.0711
100	6.1148, 6.1958, 6.2281, 6.2518, 6.2520, 6.2920	6.2224	0.0615
90	Four specimens’ lives > 6.3010		

## P-S-N curve fitting for T-welded joint

According to the analysis of fatigue test results, this article focuses on the study of P-S-N curves of T-welded joints, which based on the characteristics of crack initiation at the weld toe and crack propagation along the weld toe direction. The improved maximum likelihood method, the least square method and the maximum likelihood method are used to fit the P-S-N curves of the data in Table 3, and the P-S-N curves of F2-level in BS7608 and FAT 80 in IIW are introduced. The P-S-N curves fitted by five methods are compared and analyzed to verify the accuracy of the P-S-N curves fitted by improved maximum likelihood method. Specifically, the curves is extrapolated according to the AASHTO standard^34^.

### The least square method

**Figure 5:**
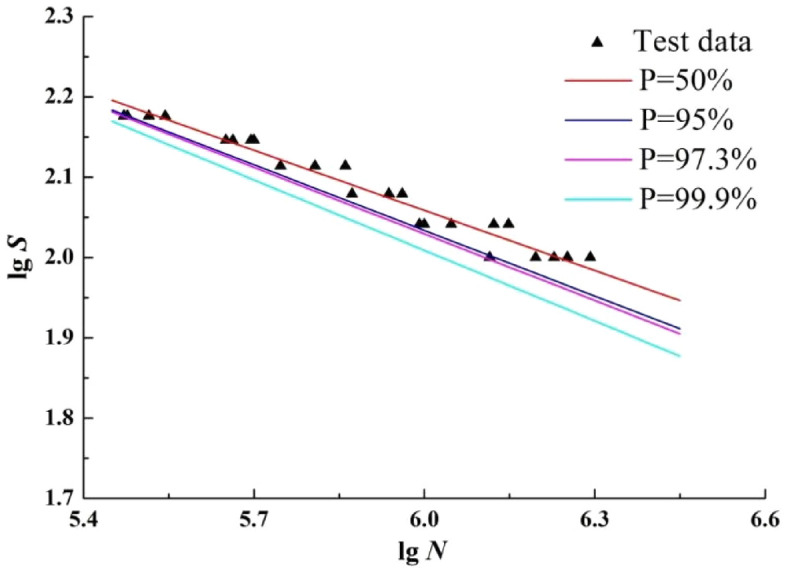
The P-S-N curves by least square method.

The least square method is used to fit the P-S-N curve for the test data in Table 3 when the survival probability is 50%, 95%, 97.3% and 99.9%, as shown in Fig. 5.

According to Fig. 5, there is no curve with high survival probability that crosses the curve with low survival probability, which is consistent with the objective law of fatigue life distribution; With the decrease of stress level and the increase of survival probability, the difference of fatigue life between the three curves gradually increases, which is consistent with the law that the dispersion of fatigue life increases with the decrease of stress level. In addition, three of the curves are basically within the range of sample fatigue life.

### The maximum likelihood method

The maximum likelihood method is used to directly fit the P-S-N curve of the data in Table 3. First, the curve equation of life mean and stress level which is fitted with the least square method as follows:

18 $$\mu_{i} = 14.1862 - 3.9766Y_{i}$$

Then, the curve equation of life std and stress level which is fitted with the least square method as follows:

19 $$\sigma_{i} = 0.5089 - 0.2199Y_{i}$$

Finally, according to the relevant parameters in Eq. (18) and formula Eq. (19), and the mean and the std on the specific stress level 110 MPa, the P-S-N curve can be obtained by maximum likelihood method as follows:

20 $$Y_{i} = \frac{{6.0616 + 0.0711u_{p} - X_{p} }}{{\left( {0.2199u_{p} + 3.9766} \right)}} + 2.0414$$

**Figure 6:**
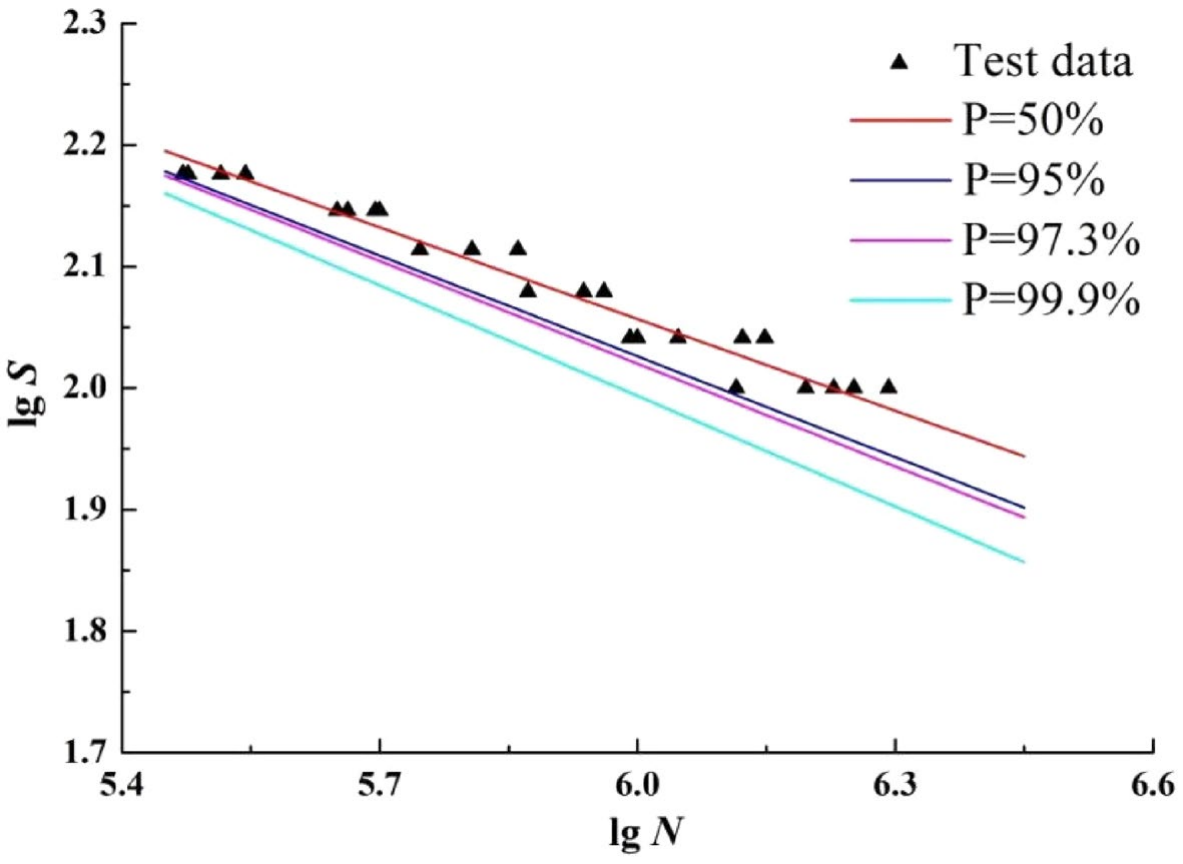
The P-S-N curves by maximum likelihood method.

According to Eq. (20), the P-S-N curves are fitted with survival probabilities *p* of 50%, 90%, 97.3% and 99.9% respectively and extrapolation, as shown in Fig. 6. For a more intuitive interpretation, the fatigue test data for the T-welded joints are plotted together in Fig. 6.

According to Fig. 6, the curve fitted by the maximum likelihood method is basically consistent with the overall characteristics of the curve fitted by the least squares method. However, only one curve is in the range of sample fatigue life, and one curve is on the boundary of the range. Compared with the least square method, the curve gradually moves down with the increase of survival probability. It shows that the P-S-N curve fitted by this method is safer than the curve fitted by the least square method.

### The improved maximum likelihood method

#### Samples information reconstruction

According to the test data in Table 3, the mean lives and stress levels follow the same approximate linear relationship in logarithmic coordinates. The line can be directly fitted by the least squares method, and the relationship between life mean and the stress level is:

21 $$\mu_{i} = 14.2562 - 4.0096Y_{i}$$

Based on Eq. (21), the mean life can be obtained for new stress levels, as shown in Table 4.

**Table 4:** Mean lives and stds for new stress levels.

Maximum nominal stress/MPa	Mean	Std
147	5.566	0.0303
125	5.8476	0.0514
105	6.1562	0.0658

According to the test data in Table 3, the stds and stress levels follow roughly the same linear relationship in logarithmic coordinates, but the stds for stress levels 150 MPa, 130 MPa, and 110 MPa are higher than those under 140 MPa, 120 MPa, and 100 MPa. Therefore, the sample information reconstruction method can be used to reconstruct the sample information for two adjacent stress levels. After the reconstruction shown in Table 4, and the relationship between std and stress level is determined to be:

22 $$\sigma_{i} = 0.5484 - 0.2384Y_{i}$$

#### Life equivalent

Based on Eqs. (21) and (22), the mean and std for each stress level can be obtained, as shown in Table 5.

**Table 5:** Mean lives and stds after information reconstruction and fitting.

Maximum nominal stress/MPa	Mean	Std
150	5.5309	0.0296
140	5.6512	0.0368
130	5.7803	0.0444
120	5.9194	0.0527
110	6.071	0.0617
100	6.237	0.0716

According to the test data in Table 3, the number of specimens for stress level 110 MPa is relatively larger, and this stress level is close to the other stress levels. Therefore, stress level 110 MPa is selected as the specific stress level. Based on the equivalent life principle, the data in Tables 3 and 5 are equivalent, as shown in Table 6. At the same time, the number of specimens under stress level 110 MPa has increased from 5 to 26.

**Table 6:** Fatigue lives under stress level 110 MPa after equivalence.

Log-life after equivalence	Mean	Std
5.9657,6.0355,6.0634,6.0838,6.0839,6.0693,6.0901,6.1438,6.1525,6.0922,6.1195,6.0243,6.1087,6.1827,6.0851,6.1426,5.9454,5.9460,5.9590,6.0378,6.0978, 5.9909,5.9997,6.0472,6.1222,6.1482	6.059	0.0676

#### P-S-N curve fitting

The P-S-N curve is fitted by the maximum likelihood method with the data in Tables 5 and 6, the formula for the curve is:

23 $$Y_{i} = \frac{{6.059 + 0.0676u_{p} - X_{p} }}{{3.9305 + 0.2772u_{p} }} + 2.0414$$

**Figure 7:**
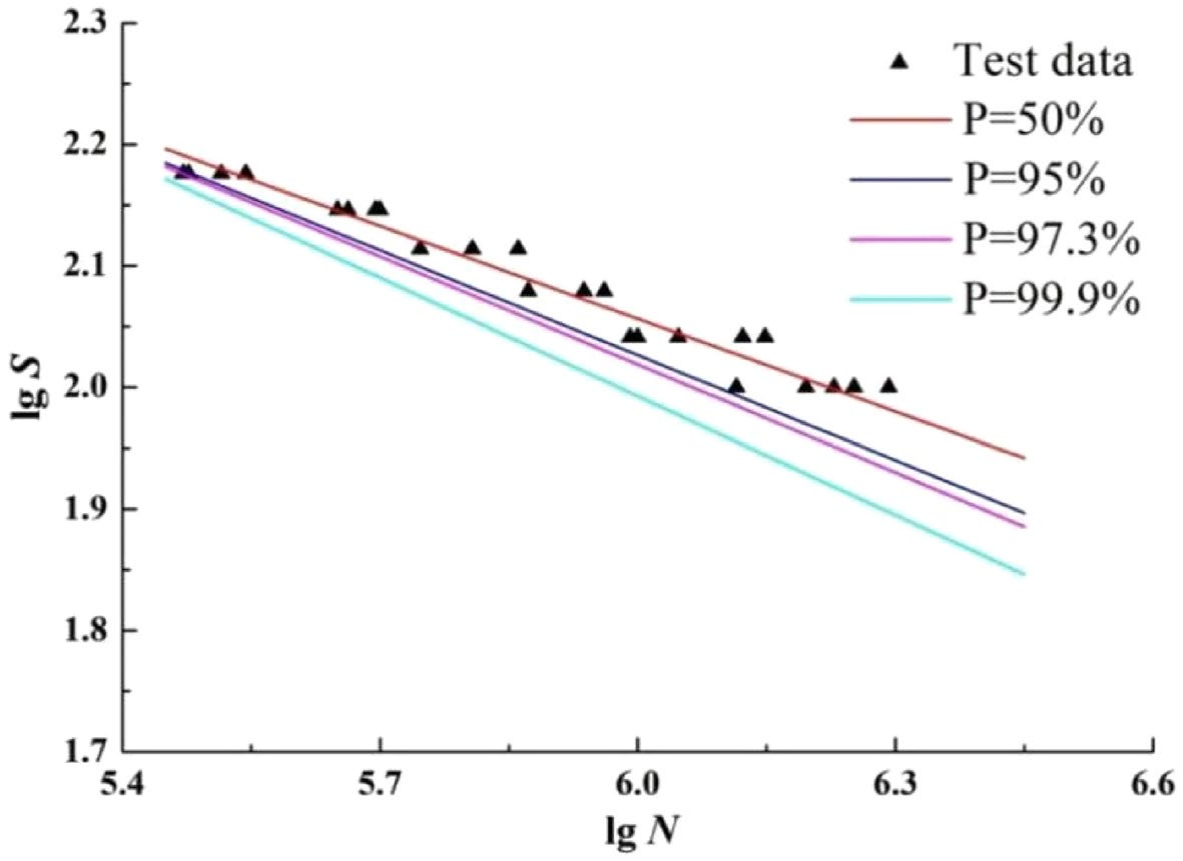
The P-S-N curves by improved maximum likelihood method.

According to Eq. (23), we fit the P-S-N curve with survival probabilities *p* of 50%, 90%, 97.3% and 99.9% respectively and extrapolation, as shown in Fig. 7.

According to Fig. 7, the curve fitted by the maximum likelihood method is also basically consistent with the overall characteristics of the curve fitted by the least squares method. There are three curves within the range of sample fatigue life. However, two of the curves are only in the low life zone and in the sample fatigue life range. With the increase of fatigue life, the curve gradually deviates from the sample life range. The reason is that the slope of the P-S-N curve fitted by this method is larger than that fitted by the maximum likelihood method.

### Comparative analysis

In order to verify the accuracy of the P-S-N curve fitted by improved maximum likelihood method, the comparison and analysis of P-S-N curves fitted by different methods and standards under different survival probabilities is as follows.

#### *Survival probabilities*—*50% and 99.9%*

**Figure 8:**
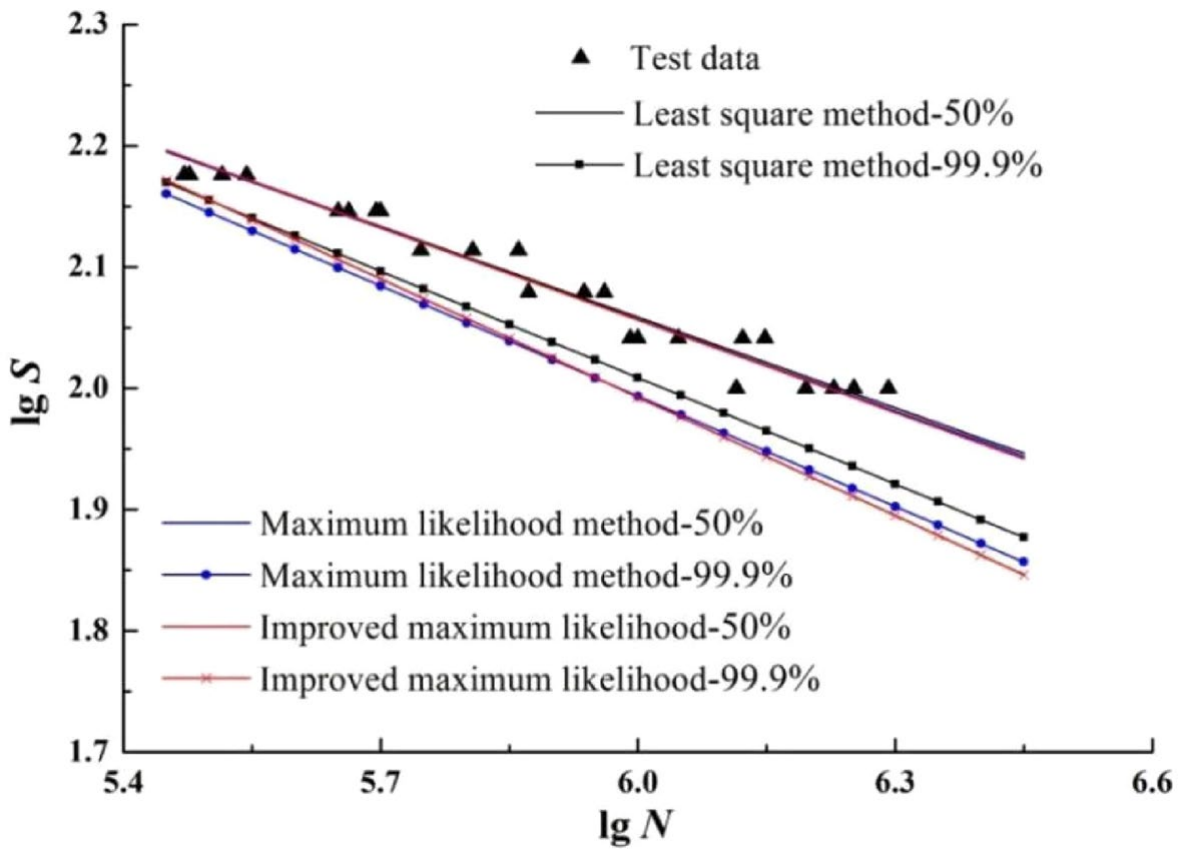
The P-S-N curves with survival probabilities 50% and 99.9%.

P-S-N curves with survival probabilities of 50% and 99.9% are extracted from Figs. 5, 6 and 7 respectively, as shown in Fig. 8.

According to Fig. 8, the conclusions that can be summarized as follows:

- The three median S–N curves basically coincide, but the P-S-N curves with the survival probability of 99.9% differ greatly. It shows that the maximum likelihood method is greatly affected by the survival probability.
- The median S–N curves are within the sample fatigue life interval, but the P-S-N curves with survival probability of 99.9% are on the left side of the sample life interval. It shows that 99.9% of P-S-N curve is more safe.
- When the survival probability is 99.9%, the slope of the P-S-N curve fitted by the maximum likelihood method is basically the same as that fitted by the least squares method, but the slope of the P-S-N curve fitted by the improved maximum likelihood method is relatively large. It shows that the improved maximum likelihood method not only optimizes the slope of P-S-N curve, but also the fitted P-S-N curve is more safe.

#### *Survival probability*—*95%*

**Figure 9:**
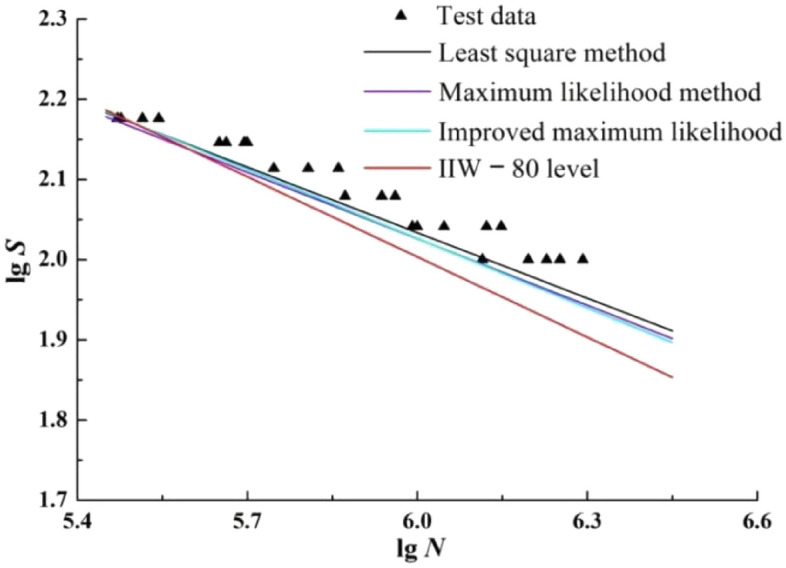
The P-S-N curves with survival probability 95%.

P-S-N curves with survival probability of 95% is extracted from Figs. 5, 6 and 7 respectively, and the S–N curve with the FAT St. 80 parameters of the T-joint in “Recommendations for fatigue design of welded joints and components^35^” (which is abbreviated to IIW**—**80 in the following) is plotted together in Fig. 9.

According to Fig. 9, the conclusions that can be summarized as follows:

- On the whole, in the high stress range, the P-S-N curves fitted by the four methods are relatively similar, but with the decrease of the stress level, the four curves gradually tend to disperse.
- Although the slope of the P-S-N curve fitted by the improved maximum likelihood method is slightly higher than that fitted by the maximum likelihood method, and the difference between the two is not obvious.
- The S–N curve slope of IIW**—**80 is greater than that of the other three curves, and the curve is safer.

#### *Survival probability*—*97.3%*

**Figure 10:**
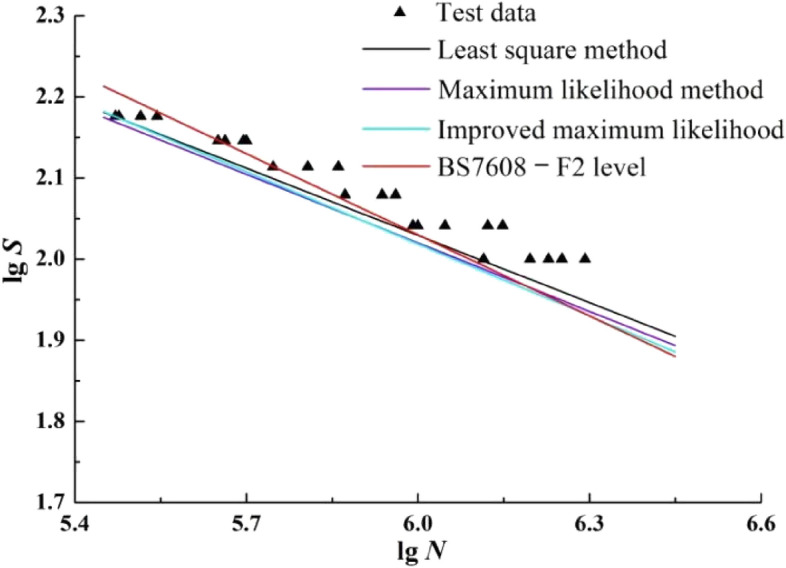
The P-S-N curves with survival probability 97.3%.

P-S-N curves with survival probability of 97.3% is extracted from Figs. 5, 6 and 7 respectively, and the S–N curve with the F2-level parameters of the T-joint in the BS7608 standard^36^ (which is abbreviated to BS7608**—**F2 in the following) is plotted together in Fig. 10.

According to Fig. 10, The S–N curve slope of BS7608**—**F2 is significantly greater than that of the other three curves. The P-S-N slope of improved maximum likelihood method is slightly larger than that of maximum likelihood method, which shows that the improved maximum likelihood method has little effect on fitting P-S-N with survival probability 97.3%.

#### *Survival probability*—*99.9%*

**Figure 11:**
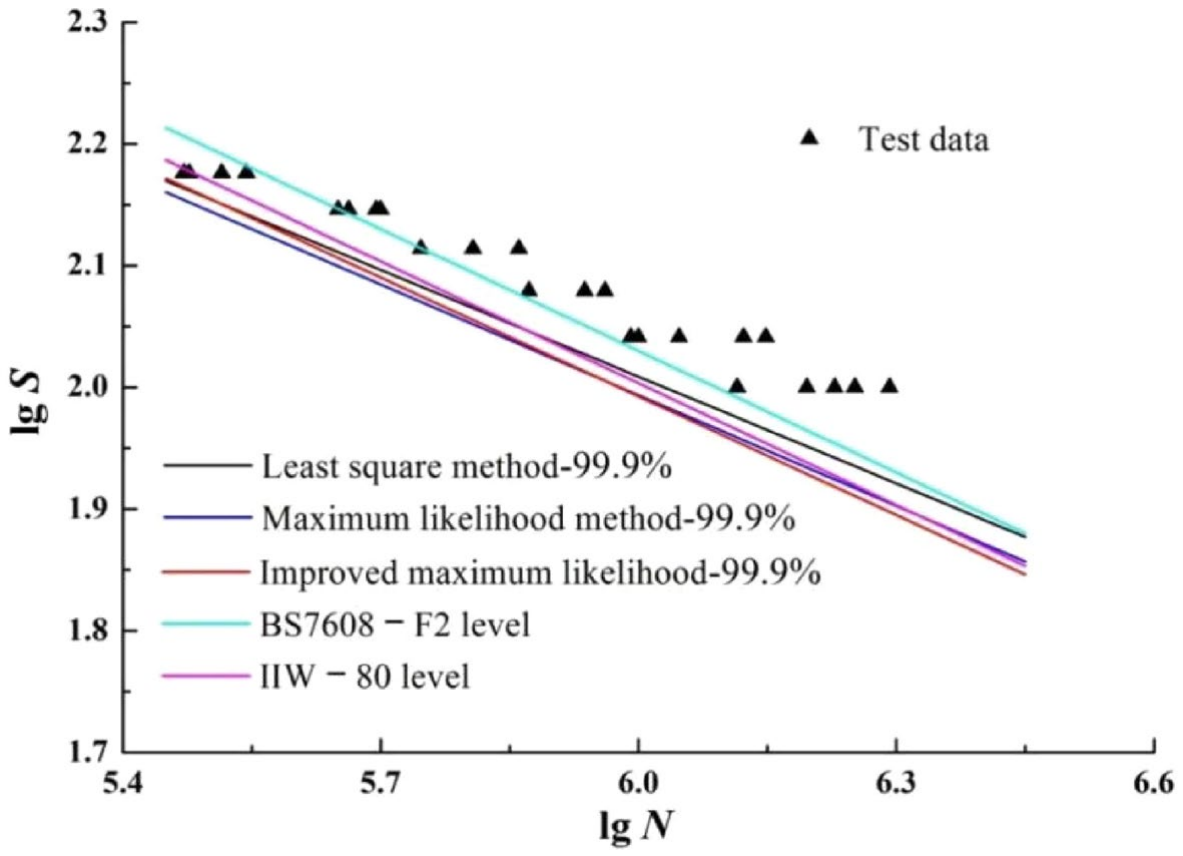
The P-S-N curves with survival probability 99.9%.

P-S-N curves with survival probability of 99.9% is extracted from Figs. 5, 6 and 7 respectively, and the S–N curves in standards of IIW**—**80 and BS7608**—**F2 are plotted together in Fig. 11.

According to Fig. 11, although the survival probabilities are different, the S–N curve slopes of improved maximum likelihood method, BS7608**—**F2 and IIW**—**80 are basically the same, and the P-S-N curve fitted by improved maximum likelihood method is the safest.

#### The key parameter value

In order to further study the effect of fitting P-S-N curve with three methods and two standards under different survival probabilities, according to Figs. 5, 6, 7, 9 and 10, the values of key parameters such as *m*, *C*_0_, and *S* (when the fatigue life is 2 × 10^6^ and 1 × 10^7^ respectively.) of P-S-N curves under different conditions are calculated, as shown in Table 7.

**Table 7:** The parameter values with different methods and different survival probability.

Survival probability (%)	Method	*m*	*S* (2 × 10^6^)	*S* (1 × 10^7^)	*C*_0_(1 × 10^7^)
50	Least square method	4.0096	96.2788	64.4466	1.7956 × 10^14^
	Maximum likelihood method	3.9766	95.7630	63.8880	1.5116 × 10^14^
	Improved maximum likelihood method	3.9305	95.4617	63.3852	1.2097 × 10^14^
95	Least square method	3.6738	89.4886	57.7431	2.9604 × 10^13^
	Maximum likelihood method	3.6149	87.6633	56.1629	2.1087 × 10^13^
	Improved maximum likelihood method	3.4745	87.0449	54.7723	1.0979 × 10^13^
	**IIW-80 level**	**3**	**80**	**46.8**	**1.024 × 10**^**12**^
97.3	Least square method	3.6206	88.3413	56.6370	2.2244 × 10^13^
	Maximum likelihood method	3.5522	86.1737	54.7766	1.4991 × 10^13^
	Improved maximum likelihood method	3.3761	85.0499	52.7995	6.5428 × 10^12^
	**BS7608-F2 level**	**3**	**85.0485**	**35**	**1.231 × 10**^**12**^
99.9	Least square method	3.4188	83.3221	52.0361	7.3753 × 10^12^
	Maximum likelihood method	3.2971	79.8284	48.9954	3.7382 × 10^12^
	**Improved maximum likelihood method**	**3.0739**	**78.4730**	**46.4863**	**1.3343 × 10**^**12**^

According to Table 7, the conclusions that can be summarized as follows:

- On the whole, under different survival probabilities, the parameter values obtained by the least squares method are the largest, and the parameter values obtained by the improved maximum likelihood method are the smallest. It shows that the P-S-N curve fitted by the least square method has the worst safety, and that fitted by the maximum likelihood method has the best safety.
- When the survival probability is 50%, each parameter value is significantly higher than the corresponding value of other survival probabilities. It shows that the median S–N curve has the worst safety and should be carefully used in fatigue life assessment.
- When the survival probability is 99.9%, the *m* of the P-S-N curve fitted by the improved maximum likelihood method are close to the corresponding values of IIW-80 level and BS7608-F2 level, which are bold in the Table 7. Meanwhile, the parameter values (except *C*_0_) of improved maximum likelihood method are generally similar with that of IIW-80 level, it shows that the P-S-N curve fitted by the improved maximum likelihood method is more safe.

Based on the above analysis, the improved maximum likelihood method was finally determined as the best method to fit the P-S-N curve. The P-S-N curve of T-type welded joints with the survival probability of 99.9% can be used in practical engineering applications.

## Conclusions

The improved maximum likelihood method was proposed for P-S-N curve fitting when only a small number of specimens is available. The fitting accuracy of the method were compared and analyzed with two methods and two standards by the T-joint specimens fatigue test data. The following conclusions can be drawn:

- The sample information reconstruction method proposed in this paper not only effectively reduces the influence of abnormal specimens and small samples on the std, but also improves the accuracy of the std at each stress level, which directly improves the accuracy of maximum likelihood method fitting P-S-N curve.
- The improved maximum likelihood method is a better way to fit P-S-N curves, which has been confirmed in the comparative analysis with other methods and standards. The accuracies of the mean lives and stds are improved by sample information reconstruction and the life equivalent principle, thus, the accuracy of the P-S-N curve is improved as well.
- The parameter values of P-S-N curve with the survival probability of 99.9% obtained by the improved maximum likelihood method are close to the corresponding values of IIW-80 level and BS7608-F2 level, and the P-S-N curve fitted by the maximum likelihood method has the best safety.
- The P-S-N curve with the survival probability of 99.9% obtained by the improved maximum likelihood method has been used in the fatigue life assessment of wagon body for three years in China. According to the feedback of technical personnel of relevant enterprises, the application effect is good.

## Data Availability

The datasets used and/or analysed during the current study available from the corresponding author on reasonable request.
